# Three-dimensional arrangement of elastic fibers in the human corneal stroma^☆^

**DOI:** 10.1016/j.exer.2015.12.006

**Published:** 2016-05

**Authors:** Philip N. Lewis, Tomas L. White, Robert D. Young, James S. Bell, C. Peter Winlove, Keith M. Meek

**Affiliations:** a Structural Biophysics Research Group, School of Optometry and Vision Sciences, Cardiff University, Maindy Road, Cardiff CF24 4HQ, UK; b Department of Physics, University of Exeter, Stocker Road, Exeter EX4 4QL, UK

**Keywords:** Elastic fibers, Microfibrils, Cornea, Pre-Descemet's layer, Ocular pulse, Trabecular meshwork, Glaucoma, TPF, Two photon fluorescence, DALK, deep anterior lamellar keratoplasty, DSEK, Descemet's stripping endothelial keratoplasty, DMEK, Descemet's membrane endothelial keratoplasty, TEM, Transmission electron microscopy, SHG, Second harmonic generation, SBF, SEM Serial block face scanning electron microscopy, HDBR, Human Developmental Biology Resource, SEM, scanning electron microscope, PEG, polyethylene glycol, PBS, phosphate buffered saline, IMS, industrial methylated spirit, TM, trabecular meshwork, IOP, intraocular pressure

## Abstract

The cornea is the main refracting lens in the eye. As part of the outer tunic it has to be resilient, a property conferred by the organisation of the constituent collagen. It also has to be sufficiently elastic to regain its exact shape when deformed, in order not to distort the retinal image. The basis of this elasticity is not fully understood. The purpose of this study was to characterise in three dimensions the arrangement and distribution of elastic fibers in the human corneal stroma, using serial block face scanning electron microscopy. We have demonstrated that there exists a complex network of elastic fibers that appear to originate in the sclera or limbus. These appear as elastic sheets in the limbus and peripheral cornea immediately above the trabecular meshwork which itself appears to extend above Descemet's membrane in the peripheral stroma. From these sheets, elastic fibers extend into the cornea; moving centrally they bifurcate and trifurcate into narrower fibers and are concentrated in the posterior stroma immediately above Descemet's membrane. We contend that elastic sheets will play an important role in the biomechanical deformation and recovery of the peripheral cornea. The network may also have practical implications for understanding the structural basis behind a number of corneal surgeries.

## Highlights

• We have characterised a complex system of elastic fibers in the posterior cornea.
• These exist as sheets in the limbus that fenestrate and become fibers in the cornea.
• We believe these fibers provide the elastic restoring force in the peripheral tissue.
• This system should help explain some surgical properties of the cornea.

## Graphical abstract

**Figure:**
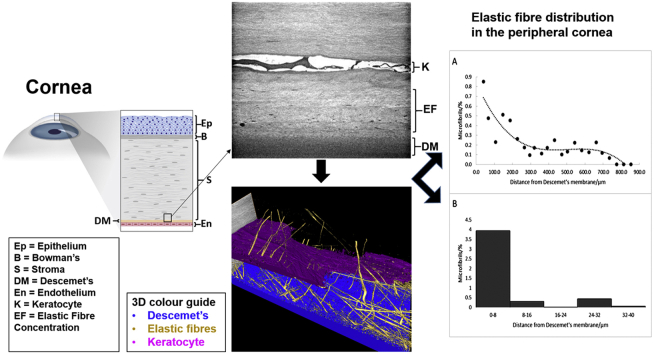

## 1 Introduction

The optical and biomechanical properties of the human cornea are largely governed by the collagen-rich stroma. This layer constitutes approximately 90% of the total thickness and comprises about two hundred stacked lamellae in the central region. Stromal transparency has long been known to arise from the spatial arrangement of the parallel collagen fibrils within each lamella (Maurice, 1957, Meek et al., 2003), which itself is governed by the association of collagen with interfibrillar proteoglycans (Lewis et al., 2010, Cheng and Pinsky, 2013). The biomechanical properties depend on the arrangement of these lamellae throughout the tissue (Aghamohammadzadeh et al., 2004, Petsche and Pinsky, 2013, Whitford et al., 2015). However, one aspect of the corneal stroma that has been somewhat neglected with respect to corneal development and biomechanics, is the presence of elastic tissue. In fact, elastic tissue was reported in the cornea as far back as 1860 (Kolliker, 1860). In 1906, M'ilroy (1906) described a distribution of elastic fibers found chiefly in the deeper layers of the peripheral human corneal stroma, near Descemet's membrane. Half a century later, Fullmer and Lillie (1958) used the term oxytalan for fibers in periodontal membranes that were resistant to acid hydrolysis. Alexander and Garner (1983) distinguished three types of ocular elastic fibers: oxytalan (precursor fibers without elastin); elaunin (intermediate elastic fibers) and true elastic fibers (with a sheath of glycoproteins surrounding an elastin-rich core). All three types of elastic fiber were present in the mature human sclera, but none were found in the normal adult cornea until 2010, when Kamma-Lorger et al. (2010) reported a network of fibers using two photon fluorescence (TPF) microscopy. These fibers were shown to run roughly parallel to the collagen lamellae in the circumcorneal annulus (Newton and Meek, 1998), a deep limbal structure that is supposed to help maintain the change in curvature between the cornea and the sclera (Abahussin et al., 2009, Boote et al., 2009). They were also seen in smaller quantities within the central stroma. Recently, Hanlon et al. (2015), using serial block face scanning electron microscopy, described the presence of microfibril bundles in the murine cornea, which they identified as being rich in the protein fibrillin, an important component of elastic fibers (Rosenbloom et al., 1993).

Recent years have seen the development of a number of surgical techniques (deep anterior lamellar keratoplasty (DALK); Descemet's stripping endothelial keratoplasty (DSEK); Descemet's membrane endothelial keratoplasty (DMEK) etc.) aimed at avoiding penetrating keratoplasty. One DALK procedure (pneuomodissection) involves injecting air into the stroma, leading to the formation of a so-called big bubble that allows easier separation of the endothelium and Descemet's membrane from the stroma. During these types of procedure, it was noticed that there were different cleavage planes, sometimes immediately above Descemet's membrane and sometimes slightly higher within the stroma (Jafarinasab et al., 2010, Mckee et al., 2011) and in 2013, Dua et al. (2013) proposed that the corneal stroma adjacent to Descemet's membrane was a distinct layer, which has different biomechanical properties to the rest of the stroma. The presence of a distinct new layer is controversial (Jester et al., 2013, Mckee et al., 2014) and it has been suggested that the plane above this so-called pre-Descemet's stromal layer is non-reproducibly determined by the variable distances of keratocytes to Descemet's membrane within and between corneas (Schlotzer-Schrehardt et al., 2015). Nevertheless these developments have highlighted the need to explore in detail the structural and hence the biomechanical properties of the corneal stroma at all positions and depths, and this must include the elastic fibers as well as the collagen lamellae.

In this paper we use conventional transmission electron microscopy (TEM), serial block face scanning electron microscopy (SBF SEM) and the nonlinear microscopy techniques second harmonic generation (SHG) and two photon fluorescence (TPF), which reveal the three-dimensional organisation of fibrous collagen and elastic fibers, respectively. Combining these techniques allows comparison of the structures seen at low magnifications (Green et al., 2014) with those seen at the high magnification electron microscope level, within the human corneal stroma.

## 2 Methods

### 2.1 Tissue specimens

Five human corneas were obtained from the CTS Eye Bank, Bristol, UK. Cornea 1 was from a 69-year–old female. The whole enucleated eye was fixed in 4% paraformaldehyde. The cornea was removed with a scleral rim and placed in modified Karnovsky's fixative (2.5% glutaraldehyde and 2% paraformaldehyde in 0.1M cacodylate buffer at pH 7.2) for 30 min, dissected and processed for serial block face scanning electron microscopy (SBF SEM) and TEM. Cornea 2 was from a 50-year-old male and was received from the Eye Bank in Eagle's minimum essential medium as a cornea with about 2 mm of the adjacent sclera. It was de-swelled with 8% dextran overnight, mounted in a Barron artificial anterior chamber to maintain a trans-corneal pressure and was fixed using the same modified Karnovsky's fixative for 3 h. It was then dissected and used for SBF SEM and TEM. Cornea 3 was from a 79-year-old male. It was collected and fixed in modified Karnovsky's fixative within two days of death and was processed for TEM using the orcein method (see below). Cornea 4 was from a 72-year old female. It was fixed in 4% paraformaldehyde then wax embedded for histology. Cornea 5 from a 67-year-old male was processed for non-linear microscopy as described below. Cornea 6 was from a 13-week old foetus obtained from the Human Developmental Biology Resource (HDBR). The whole globe was fixed in modified Karnovsky's fixative and was then processed for SBF SEM as below. Institutional Ethics Committee approval was obtained for this study and the research followed the Tenets of the Declaration of Helsinki.

### 2.2 Serial block face scanning electron microscopy

Human Corneas were fixed for 3 h in 2.5% glutaraldehyde/2% paraformaldehyde in 100 mM sodium cacodylate buffer pH 7.2 at room temperature (RT). The cornea was cut into thin segments and post fixed with 1% osmium tetroxide for 1 h. After a brief wash with fresh buffer followed by a 20 min wash in distilled water the samples were incubated in 0.5% low molecular weight (di-Gallic) C_14_H_10_O_9_ tannic acid (mw 322.22) (AR Mallinckrodt, Dublin, Ireland) in distilled water for 2 h. The samples were then washed in distilled water for 30 min and placed in 1% aqueous uranyl acetate for 1 h in the dark at RT. Corneal samples were then dehydrated in an ethanol series from 70% to 100% ethanol for 1 h after which they were placed in 2% uranyl acetate in 100% ethanol for 2 h in the dark at RT. After washing in 100% ethanol for 40 min, the corneal samples were placed in a 1:1 mixture of 100% ethanol: 100% acetone for 20 min. The samples were then incubated in a saturated solution of lead acetate in a mixture of 1:1 100% ethanol 100% acetone for 2 h (Kushida, 1966). After staining, the corneal samples were washed in two changes of 1:1 mixture of 100% ethanol and 100% acetone for 15 min and placed in 100% solution of acetone for 20 min. After washing the samples 3 times for 20 min in 100% acetone, the samples were finally embedded in CY212 (TAAB) epoxy resin and polymerised for 24 h at 60° C.

The surfaces of polymerised resin blocks were then trimmed and attached to Gatan (PEP6590) specimen pins. The pins were then gold coated and transferred to a Zeiss Sigma VP FEG SEM equipped with a Gatan 3View2 system, where data sets of up to 1000 images were acquired of the block surface every 50 nm through automated sectioning. Each image was acquired at 4K × 4K pixels, at a pixel resolution of 4 nm and a pixel dwell time of 8 μs, using an accelerating voltage of 3.4 keV in low vacuum variable pressure mode (28 Pa). Imaging data was acquired from a 16.19 μm × 16.19 μm region of interest. Selected serial image sequences were extracted from the image data and 3D reconstructions were generated with Amira 6.0 software (FEI, Mérignac, France) using both manual hand tracing for the larger structures and automated thresholding for the fibers.

For the full thickness quantification analysis, the peripheral cornea was imaged every 50 nm en face at 5000 × magnification, starting from the epithelium and ending at Descemet's membrane, obtaining over 21,000 images with resolutions of 2000 × 2000 pixels. The data were split into sets of ∼1000 images, fibers were segmented using a mixture of manual and automatic thresholding, resulting in a fiber voxel count that was subsequently converted to percentage using the total volume voxel count. Cells were blocked out to background level in order to remove their contribution to the images. The more detailed quantification above Descemet's membrane was carried out by measuring fiber volume every 200 images. Both were done using Amira 6.0 software with XImagePAQ extension. When sectioning the 16 μm × 16 μm block face through a thickness of almost 1 mm, it was very difficult to ensure that the en face sections were exactly parallel to the surface of the cornea. When viewing our series of images from different depths, it was evident that the blocks were being cut at a slight angle to the surface of the cornea. To correct for this, the number of 50 nm sections between where Descemet's membrane was just visible down to the depth where the stroma was just not visible (which ideally would be zero if the block was being cut parallel to the corneal surface) was used to make a geometrical calculation of the offset angle and hence correct the section thickness and the depth measurements to allow for this when plotting the % fiber volume as a function of depth. This led to an uncertainty of ±5 μm in each depth measurement.

### 2.3 Transmission electron microscopy

Embedded samples from Corneas 1 and 2 that had been en bloc stained for SBF SEM, were also used for TEM. A Leica UC6 ultra-microtome was used to cut 90 nm-thick gold sections that were floated on distilled water before being mounted on copper grids. Additionally, separate sections were cut from Cornea 3 and stained with the elastic fiber-specific stain 0.2% orcein in acid alcohol (1% HCl in 70% EtOH), then rinsed with 70% EtOH, air-dried, and counterstained with uranyl acetate and lead citrate. Both normal and orcein stained ultra-thin sections were visualised using a Jeol 1010 TEM (JEOL, Tokyo, Japan).

### 2.4 Non-linear microscopy

Corneal buttons 8 mm in diameter were dissected from the centre of Cornea 5, placed into dialysis tubing (molecular cut-off 14 kDa), and brought to physiological hydration by immersion in 2.5% polyethylene glycol (PEG) overnight. The buttons were then mounted on Superfrost glass slides, in 1:1 phosphate buffered saline (PBS) – glycerol solution, protected by a 0.16 mm thick glass coverslip. A modified confocal microscope (Olympus FluoView IX71 and F300) was used to obtain TPF and SHG images (see Bell et al. (2014) for a complete description of the system configuration). TPF and SHG images from the same area of cornea were overlaid using ImageJ software (Schneider et al., 2012), resulting in a composite image.

### 2.5 Histology

Paraffin wax sections were dewaxed in xylene and rehydrated in a descending industrial methylated spirit (IMS) gradient to water. Tissue sections were incubated in Miller's elastic stain at 60 °C for 1 h, destained in 95% IMS and washed in water before 10 min of nuclear staining in Mayer's haematoxylin, followed by counterstaining of collagen fibrils with Van Gieson's stain for 5 min. Sections were then washed in water, dehydrated through an ascending IMS gradient, cleared in xylene and mounted under coverslips with DPX mountant. Elastic fibers and collagen appeared purple/black and pinkish/red, respectively.

## 3 Results

**Fig. 1:**
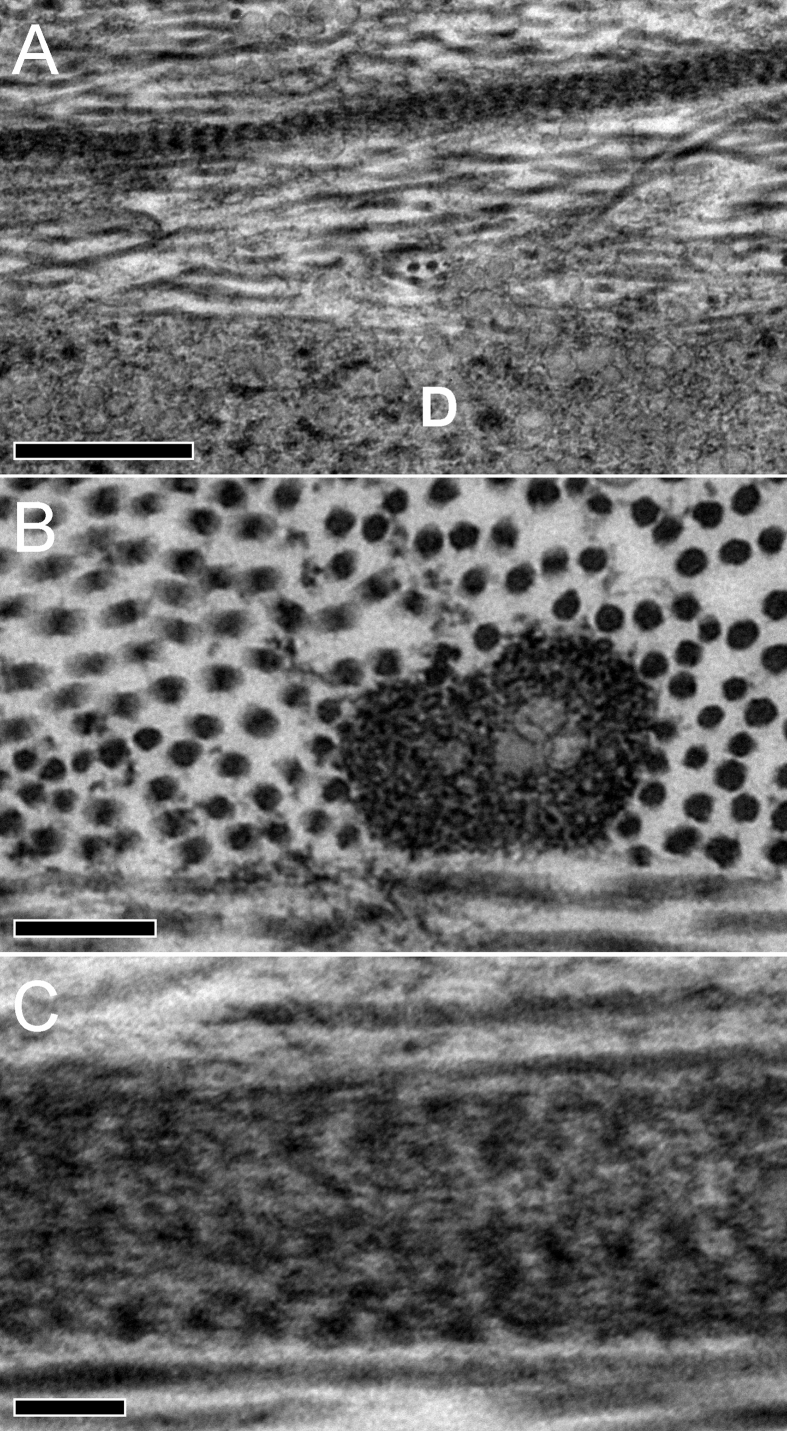
TEM images of elastic fibers from the central cornea (Cornea 1) stained with tannic acid. Fig. 1A: A banded fiber running longitudinally above Descemet's membrane (D) (bar = 500 nm). Fig. 1B: In cross-section, the elastic fibres show a substructure, with less intense staining in the center (bar = 200 nm). Fig. 1C: At high magnification some elastic fibers shows less distinct banding and longitudinal texture suggesting a microfibrillar substructure (bar = 100 nm).

Ultrathin sections examined using TEM revealed elastic fibers that were clearly distinct from the collagen fibrils (Fig. 1). The tannic acid-based staining method employed (the same as was used for SBF SEM) is purported to stain elastic fibers as well as collagen (Simmons and Avery, 1980, Kageyama et al., 1985), and the elastic structures were thus heavily stained and appeared as short fibers or dark irregular structures in cross-section. When viewed longitudinally, they exhibited banding with a pseudo-periodicity of 56.5 ± 4.8 nm (Fig. 1A). They were also revealed to contain numerous smaller structures, possibly individual microfibrils (Fig. 1B) and exhibited a textured longitudinal substructure (Fig. 1C). The widths of the individual elastic fibers were highly variable but most were found to lie between 100 nm and 200 nm although some, particularly in the peripheral stroma, were larger, up to 500 nm.

**Fig. 2:**
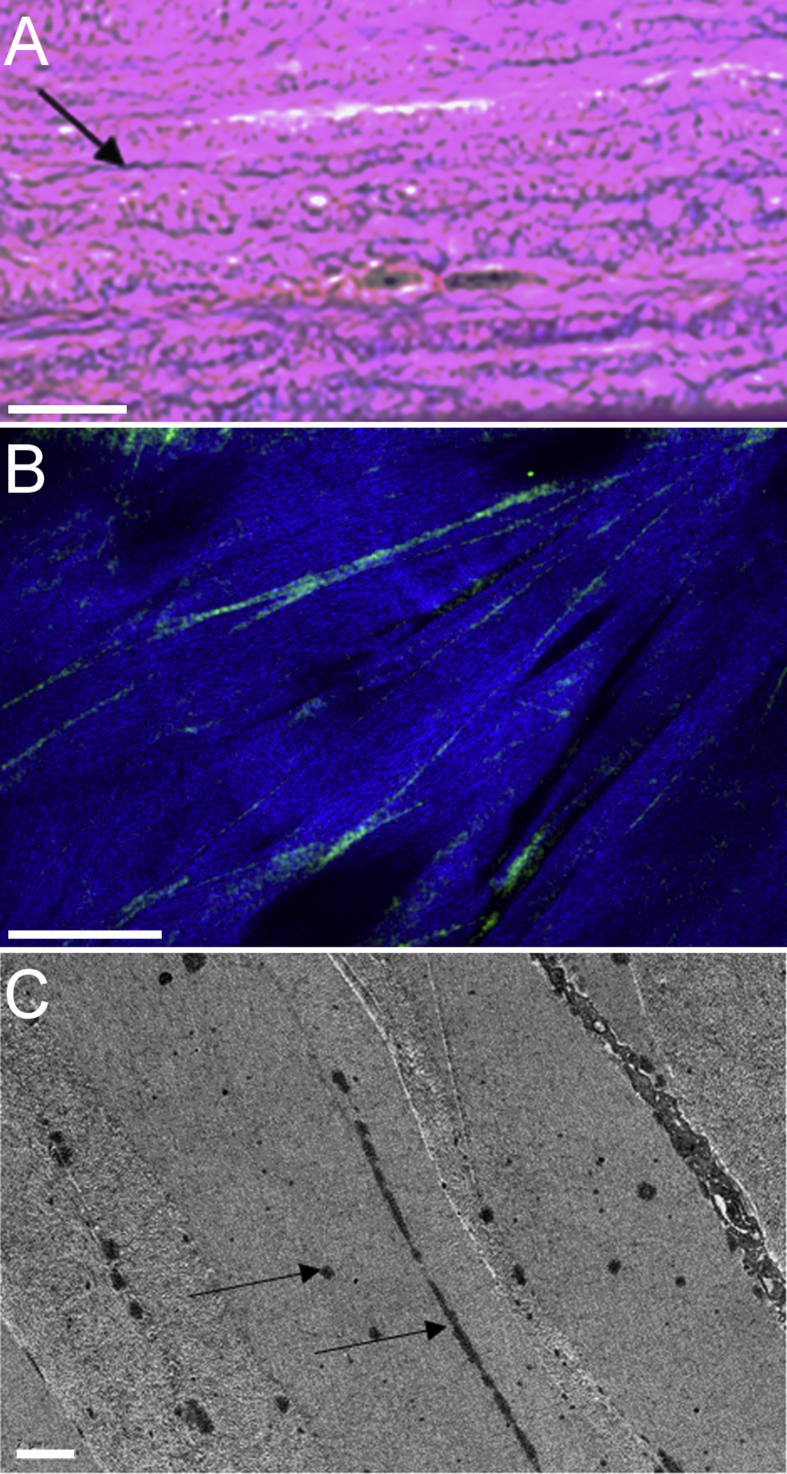
Images of the peripheral stroma (4–5 mm from the optic axis) stained for elastic fibers. Fig. 2A: Transverse histological section (Cornea 5) immediately above Descemet's membrane, stained with Miller's elastic stain. Numerous elastic fibers are stained black/purple (arrow), particularly in the most posterior stromal lamellae at the bottom of the image. Bar = 10 μm; Fig. 2B: En face combined two photon fluorescence (TPF) and second harmonic generated (SHG) image (Cornea 4). Elastic fibers are shown in green (TPF) and collagen lamellae in blue (SHG). Bar = 50 μm; Fig. 2C: Transverse TEM image of posterior stroma using orcein staining for elastic fibers (Cornea 3). The dark electron dense fibers (black arrows) are easily distinguishable from background stromal collagen. The fibers are present both within individual lamellae and between lamellae. Bar = 2 μm. (For interpretation of the references to colour in this figure legend, the reader is referred to the web version of this article.)

To confirm the nature of the elastic fibers, they were stained for histology using Miller's elastic stain (Fig. 2A), examined by TPF microscopy (Fig. 2B) and visualised at the electron microscopical level, this time using the elastic fiber-specific stain orcein (Fig. 2C) (Nakamura et al., 1977). These techniques confirmed that the tannic acid-based SBF SEM staining was able to stain elastic fibers and their constituent microfibrils successfully. However, because TEM is essentially a two-dimensional technique, it was not possible to track the course of the fibers in and out of the plane of the images.

**Fig. 3:**
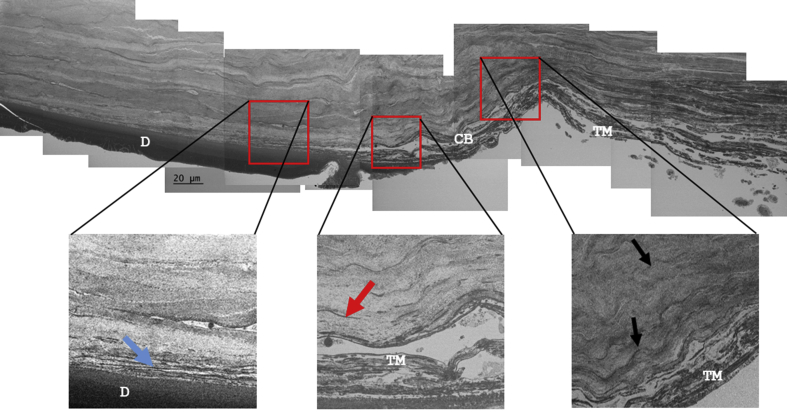
Low magnification montage of SEM images showing a transverse section through the corneolimbal region from Cornea 2. Numerous elastic structures are evident within the corneolimbal region (black arrows). The trabecular meshwork (TM) appears to have a “wedge like” insertion into the adjacent posterior cornea above Descemet's membrane (D) (blue arrow) which tapers to a terminal point some 250 μm in from the corneolimbal boundary (CB). The elastic fibers are visible above this insertion (red arrows). (The full three-dimensional structure is seen in Video clip S1). (For interpretation of the references to colour in this figure legend, the reader is referred to the web version of this article.)

**Fig. 4:**
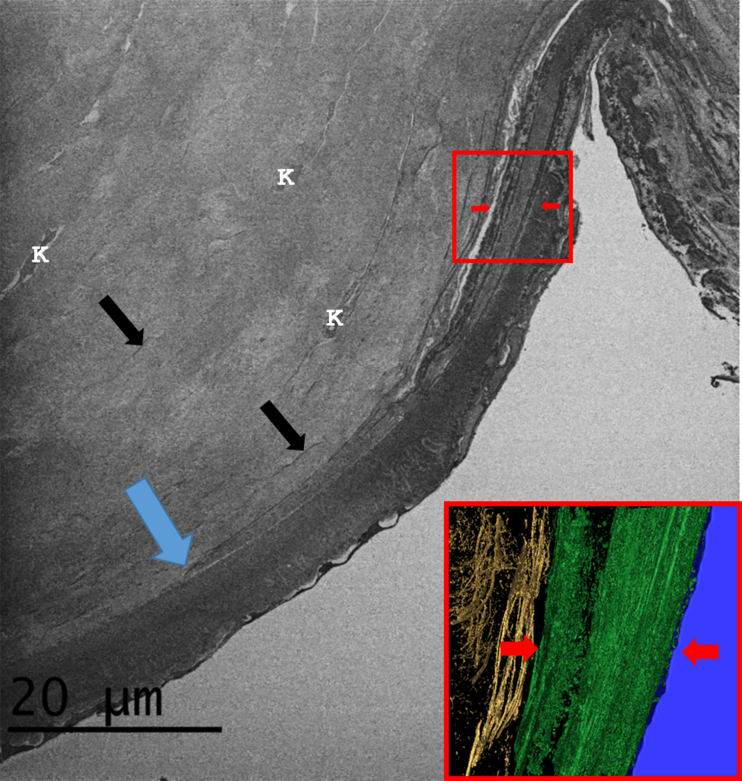
The corneolimbal region (Cornea 1). The main SEM image shows the trabecular meshwork insertion between Descemet's membrane and the posterior corneal stroma. The trabecular meshwork insertion is separated from the stroma and Descemet's membrane by sheets of elastic-like tissue (red arrows), which appear to terminate at a point (blue arrow) within the posterior stroma next to Descemet's membrane. Elastic fibers and elastic sheets are evident (black arrows) within the posterior stroma to a depth of about 25 μm distal to Descemet's membrane. Keratocytes (K) are also visible within the posterior stroma. Inset: A 3D image slice through the trabecular meshwork insertion highlighting the presence of elastic sheets (coloured green and indicated by red arrows) and fenestrated elastic sheets present within the posterior stroma above it (coloured gold). (The full three-dimensional structure is seen in Video clip S2). (For interpretation of the references to colour in this figure legend, the reader is referred to the web version of this article.)

Low magnification SBF SEM examination of the posterior corneolimbus and the adjacent cornea in transverse section revealed the presence of numerous electron-dense elastic fibers within the posterior limbus, which extended into the posterior cornea (Fig. 3). Most limbal fibers were concentrated within a 40–50 μm region of the posterior limbus anterior to the trabecular meshwork (Fig. 3 inset and Video clip S1). These fibers appeared densely packed and were generally found to be aligned parallel to the limbal stroma. At the corneolimbal junction where Descemet's membrane terminates, the fibers were less concentrated and appeared to fan out between the thicker lamellae of the posterior corneal stroma. SBF SEM of the posterior corneolimbus and the adjacent cornea also revealed that the trabecular meshwork makes a wedge-like insertion into the cornea, between Descemet's membrane and the posterior stroma (Fig. 3, Fig. 4). This TM insertion was identified to taper to a point distal to Descemet's membrane, approximately 250 μm beyond what is currently considered the posterior corneolimbal junction. The junction is currently defined as the point where Descemet's membrane terminates in the peripheral cornea and the TM is anchored anteriorly to the limbus. SBF SEM further revealed that the TM insertion is bordered by 2–4 layers of elastic sheets (Fig. 4 and Video clip S2). Anterior to the TM insertion, numerous fibers and elastic sheets that had started to fenestrate were evident (Video clip S2). These elastic sheets extended just beyond the posterior corneolimbal junction, where they appeared to transform into fiber-like extensions, which continued into the posterior corneal stroma (Video clip S2).

Supplementary video related to this article can be found at http://dx.doi.org/10.1016/j.exer.2015.12.006.

The following are the supplementary data related to this article:

Video clip S1 Related to Figure 3. Rendered three-dimensional video of the corneo-limbal region near the trabecular meshwork of a cornea (Cornea 2) excised from the eye and then fixed under intraocular pressure.

Video clip S2 Related to Figure 4. Rendered three-dimensional video of the corneo-limbal region of a cornea (Cornea 1) taken from a whole fixed eye.

**Fig. 5:**
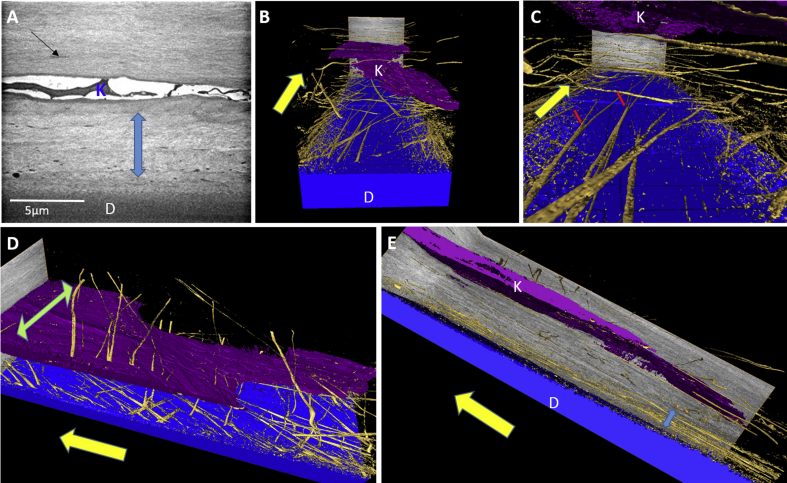
The peripheral cornea (Cornea 1). The yellow arrows indicate the radial direction towards the limbus. Fig. 5A: SBF SEM image of posterior peripheral cornea reveals the presence of numerous elastic fibers, which appear highly concentrated within a zone containing 4–5 lamellae (blue arrow) next to Descemet's membrane (D). Elastic fibers are also evident above this zone (black arrow) above a keratocyte (K). Fig. 5B: 3D volume rendering of the posterior peripheral cornea reveals the distribution of elastic fibers (gold) above and below a keratocyte (K, coloured purple) running parallel to Descemet's membrane (coloured blue). Fig. 5C: Fibers exhibit bifurcated and trifurcated branching (red arrows). Fig. 5D: The elastic fibers appear randomly orientated. Some are orientated at an oblique angle toward the limbus while others appear to be orientated more circumferentially (green arrow) with respect to the cornea. Fig. 5E: Side plane view of 3D volume reveals the elastic fibers to be concentrated just above Descemet's membrane (blue arrow). The full three-dimensional structure is seen in Video clip S3. (For interpretation of the references to colour in this figure legend, the reader is referred to the web version of this article.)

Detailed SBF SEM volume analysis of a 16 × 16 × 50 μm region of the peripheral (4–5 mm from the optic axis) posterior corneal stroma (Fig. 5A), revealed the presence of elastic fibers throughout most of the volume. These fibers were found to be densely packed within four or five thin lamellae anterior to Descemet's membrane (Fig. 5B and Video clip S3). Anterior to this concentrated 8 μm lamellar zone, they generally traced within and between the collagen lamellae. All of the elastic fibers identified were aligned approximately parallel to the plane of the stromal lamellae though some appeared to curve out of this plane (Fig. 5C). Automated iso-surface 3-D rendering of the fibers revealed them to exhibit bifurcated and trifurcated branching (Fig. 5C). Fiber orientation appeared random, with some orientated at an oblique angle to the direction of the limbus and others arranged circumferentially around the cornea (Fig. 5D). Many fibers were also found to span the entire length of the volume (Fig. 5D–E).

Supplementary video related to this article can be found at http://dx.doi.org/10.1016/j.exer.2015.12.006.

**Fig. 6:**
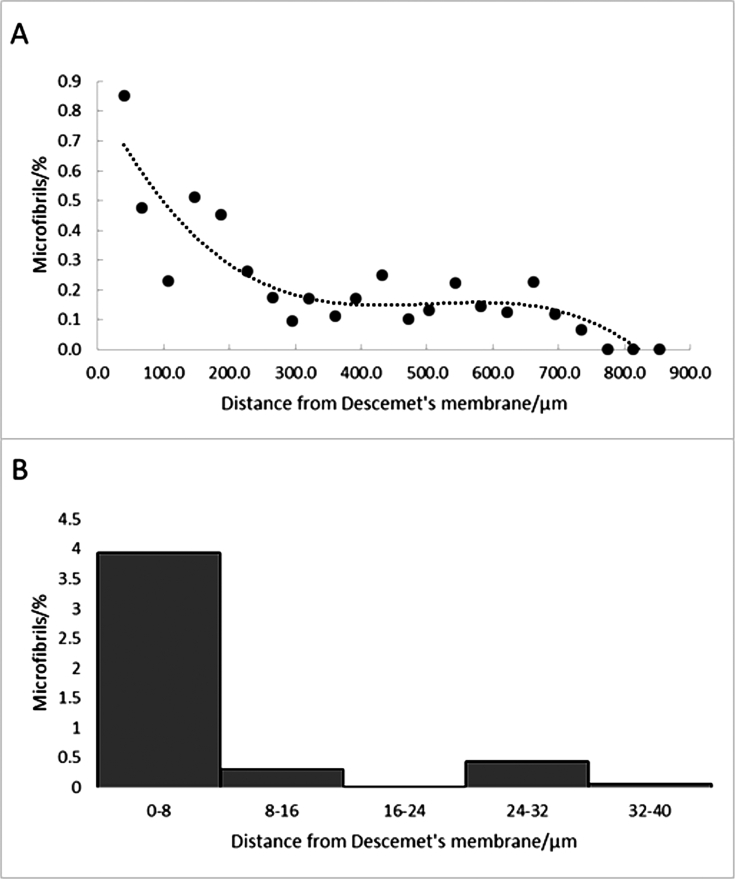
Distribution of elastic fibers as a function of depth in the peripheral cornea measured from en face images through the cornea (Cornea 1). The top graph shows the percentage of the tissue volume occupied by elastic fibers from 0 μm (top of Descemet's layer) to about 800 μm (bottom of epithelium). Each point represents data from about 1000 en face serial images summed between that depth and the one before it in the graph. A third order polynomial is included to show the main trends in the data. The number of elastic fibers falls rapidly after about 200 μm from Descemet's membrane. No fibers were observed in the anterior ∼100 μm of the stroma). Most of the fibers were seen in the first 40 μm above Descemet's membrane, so this region was measured at higher resolution. The bottom histogram shows the volume percentage of elastic fibers in this region. Each bin corresponds to 199 en face images. This shows that the majority of the fibers are located in the ∼8 μm region immediately above Descemet's membrane.

The following is the supplementary data related to this article:

Video clip S3 Related to Figure 6. Rendered three-dimensional video of the peripheral posterior stroma of a cornea (Cornea 1) taken from a whole fixed eye.

Over 21,000 en face serial images (at 50 nm intervals) were acquired through the full thickness of the peripheral cornea from Descemet's membrane to the corneal epithelium. These were divided into groups of about 1000 images, each group representing a sampled volume of about 12,800 μm^3^. All the volume elements were rendered and the percentage of the volume element occupied by elastic fibers was calculated. The results indicated that the fiber density was greatest in the posterior stroma, remained uniform 200–700 μm from Descemet's membrane, and then fell to zero in the anterior stroma and epithelium (Fig. 6A). We observed that the highest concentration appeared to be immediately above Descemet's membrane so we studied this region in greater detail (Fig. 6B). This showed that the concentration was highest in the posterior 8 μm, and fell significantly outside that region.

**Fig. 7:**
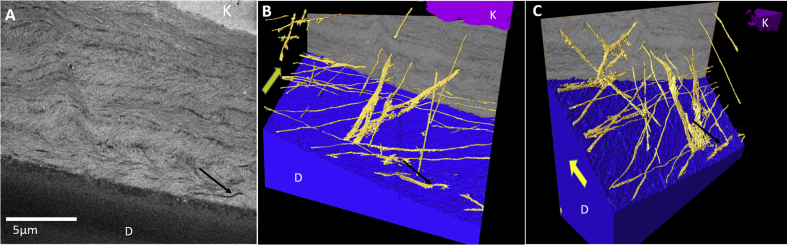
The central cornea (Cornea 1). Fig. 7A: SBF SEM image of posterior central cornea. The black arrow highlights a single elastic fiber running longitudinally within the stroma. (D = Descemet's membrane; K = keratocyte). Fig. 7B and C: Three-dimensional volume rendering of the same region shown in A reveals that most fibers run radially toward the limbus (yellow arrow). The full three-dimensional structure is seen in Video clip S4. (For interpretation of the references to colour in this figure legend, the reader is referred to the web version of this article.)

3-D volume rendering of a 16 x 16 × 50 μm region of the central posterior corneal stroma revealed that most of the elastic fibers were sparsely distributed between the stromal lamellae (Fig. 7A). No branching was evident and they appeared straight and thinner that those seen in the peripheral stroma. The lamellae above Descemet's membrane, however, were found to contain numerous fibers orientated radially and obliquely toward the limbus, with some running parallel to the anterior surface of Descemet's membrane (Fig. 7B, C and Video clip S4).

Supplementary video related to this article can be found at http://dx.doi.org/10.1016/j.exer.2015.12.006.

The following is the supplementary data related to this article:

Video clip S4 Related to Figure 7. Rendered three-dimensional video of the central posterior cornea (Cornea 1) taken from a whole fixed eye.

**Fig. 8:**
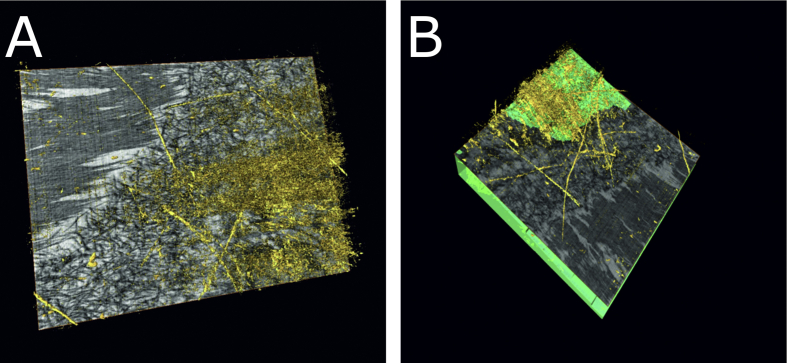
SBF SEM from the central posterior cornea of a 13-week-old human foetus (Cornea 6). Fig. 8A shows the presence of elastic fibers (gold) above a meshwork of dark filaments which will later become Descemet's membrane. Because of the oblique imaging plane, some stroma above the fibers is seen at the top left of the image. Fig. 8B, acquired from a different orientation shows elastic fibers (gold) immediately above the endothelium (light green). The full three-dimensional structure is seen in Video clip S5. (For interpretation of the references to colour in this figure legend, the reader is referred to the web version of this article.)

In order to investigate whether this system of elastic fibers differs between developing and adult human cornea, we examined a 13-week old human foetal cornea. We found that, even at this early developmental stage, there was a layer of elastic fibers, about 8 μm thick, immediately above the rudimentary Descemet's membrane and endothelium. (Fig. 8 and Video clip S5).

Supplementary video related to this article can be found at http://dx.doi.org/10.1016/j.exer.2015.12.006.

The following is the supplementary data related to this article:

Video clip S5 Related to Figure 8. Rendered three-dimensional video of the central posterior corneal stroma from a 13-week-old human foetus.

## 4 Discussion

This study reveals the presence of a highly complex elastic fiber sub-structure in the human cornea. The SBF SEM protocol used here is known to stain both the microfibrillar and the amorphous component of elastic fibers (Simmons and Avery, 1980, Kageyama et al., 1985). The fibers also stained positively in the electron microscope with the elastic-fiber stain orcein, with a histological elastic fiber stain (Miller's elastic stain), and were visualized using two photon fluorescence microscopy tuned close to the elastin two photon excitation cross-section peak. At present, the exact combination of molecules within the elastic fibers we have described in different regions the cornea is not clear, but by analogy with the mouse cornea (Hanlon et al., 2015) it is likely that they contain fibrillin microfibrils. The elastic fibers in the peripheral cornea exhibited clear banding with a periodicity of just below 60 nm, typical of fibrillin microfibrils (Jensen et al., 2012, Sherratt et al., 2003), with similar though less well-defined banding in the central cornea. This banding was not described in fibrillin-rich mouse elastic microfibril bundles (Hanlon et al., 2015). There is still much missing information about the precise organisation of the fibrillins, their association with cell matrix components, and the mechanisms of microfibril elasticity (Jensen et al., 2012). In addition to fibrillin, a number of additional molecules are known to be present in elastic fibers in other tissues (Rosenbloom et al., 1993, Marshal, 1995) and this may also be the case in the cornea. Unfortunately, we were not able to obtain immunofluorescent labelling of fibrillin 1 using several different commercially available antibodies (results not shown) but we obtained labelling with type VI collagen antibody. Type VI collagen has been shown to be abundant in the pre-Descemet's layer (Dua et al., 2014) and it is possible that its association with these microfibrils (Everts et al., 1998), or the presence of other molecules, masks the fibrillin antigenic sites in the mature human cornea. It will be important in future to identify the exact composition and structure of these elastic structures in the cornea.

**Fig. 9:**
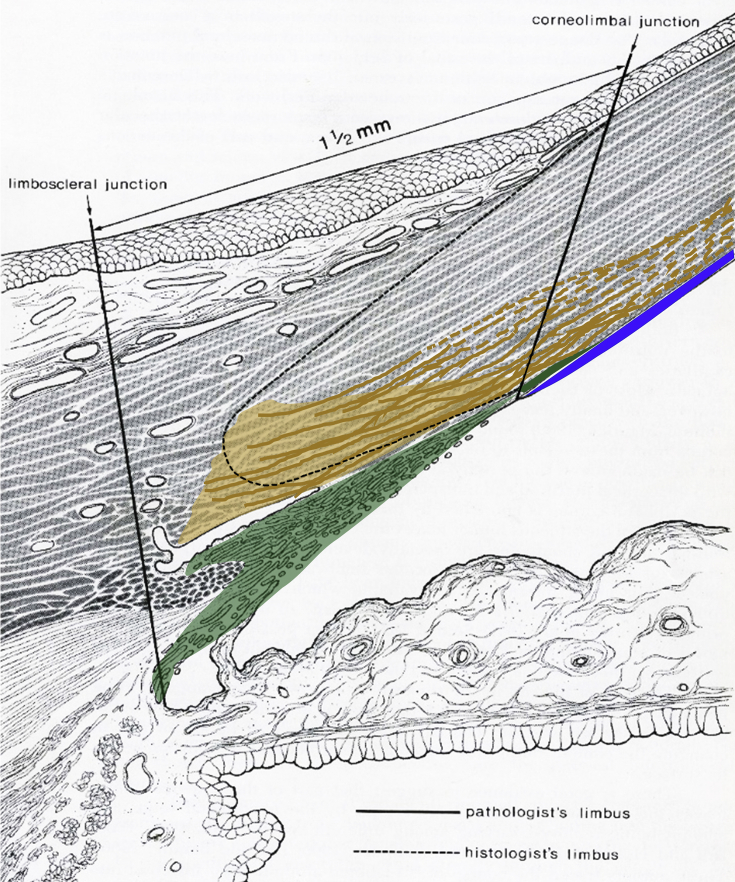
Proposed elastic fiber organisation in the limbal region. Translucent green: Trabecular meshwork. Solid green: Trabecular meshwork insertion. Gold: Elastic limbal region with concentrated elastic fiber sheets. Most elastic fibers appear to emanate from this region and extend across the posterior stroma. Continuous elastic sheets are represented by solid gold lines; transition of sheets to fibers is represented by dashed gold lines. Blue: Descemet's membrane. (Modified from Hogan et al.)^39^. (For interpretation of the references to colour in this figure legend, the reader is referred to the web version of this article.)

In the human cornea, we identified elastic fibers that are similar to the microfibril bundles found by Hanlon et al. (2015) in the mouse. However, with the range of techniques used here, in the peripheral cornea we found many that were considerably wider than this. From the SEM results many large fibers originate deep within the posterior limbus, in a 40–50 micron zone limited by Schlemm's canal and the trabecular meshwork, which itself is rich in elastic fibers (Hann and Fautsch, 2011). This elastic zone is readily identified by the presence of a heavily-stained, electron-dense region of concentrated elastic fibers that appears to be limited to the scleral spur. Some fibers are derived from compact fenestrated elastic sheets originating from the posterior limbus while others start out as broad fibers. TEM montaging of the full thickness of the peripheral cornea confirm this distribution (data not shown), reinforcing our view that many elastic fibers, even those in the central cornea, have their origins in the posterior limbus and possibly the adjacent posterior sclera and appear to continue into the cornea then back to a different part of the limbus, since no obvious fiber termination points were evident. Fig. 9 is a diagrammatic representation of these results.

Three-dimensional SBF SEM volume reconstructions of the periphery and central-posterior cornea revealed elastic fibers anterior to Descemet's membrane concentrated within a zone of about four lamellae (Fig. 6, Fig. 7). Anterior to this zone they were mainly distributed between lamellae. Fiber density data from SBF SEM full thickness analysis of the peripheral cornea show that the elastic fibers are present at all levels except the most anterior stroma, but that they are more abundant in the posterior 200 μm and are concentrated in the posterior ∼8 μm of the stroma (Fig. 6). It should be noted, however, that the threshold value chosen to highlight the elastic fibers was such as to avoid staining other tissue components, so we would not have included any weakly stained elastic fibres; the quantitative data presented should therefore be regarded as minimum values.

Elastic fibers were identified in a 13-week embryonic cornea within four very thin lamellae of the posterior stroma, forming an 8 μm thick region anterior to the endothelium.

Their presence at this early stage of development, before Descemet's membrane has formed, indicates that these fibers are not the result of degenerative age-related changes, but presumably fulfil instead some basic function in the presumptive stroma.

Our study has revealed that the TM projects about 250 μm into the peripheral cornea between the Descemet's membrane and the posterior stroma. This result confirms a recent observation by Dua et al. (2014) in which the TM was also identified to extend into the peripheral cornea, although these authors did not describe the anatomy of the TM insertion in any detail. The anatomical description of the TM in this study is very different from the currently accepted description of TM anatomy in terms of its anterior fixation. Hogan et al. (1971) describe the TM joining the limbus at a point where Descemet's membrane terminates, referring to this as the posterior corneolimbal junction (see Fig. 9). The TM is regarded as a “toned” tissue, which is required to be under tension in order to function normally. The aqueous outflow is regulated by the contraction and relaxation of the trabecular meshwork, through the action of ciliary muscles attached to the meshwork at its posterior fixation point deep within the sclera (Llobet et al., 2003, Tamm, 2009). The TM insertion would appear to be closely associated anteriorly with the concentrated elastic fiber zone identified in this study, which spanned the limbus and the peripheral cornea. It is possible that this elastic system helps maintain the tone by fixing the TM anteriorly to the peripheral cornea and limbus.

To maintain focus in vision, the cornea must be resistant to deformation from external forces, and when it is deformed it must regain its original shape quickly. In vivo, a force balance exists between the collagen network, which resists only tension, the proteoglycan gel, which exerts an internal swelling pressure on the collagen network, and the intraocular pressure (IOP). The mechanical response to changes in IOP, either over the cardiac cycle or as a symptom of disease, has been a topic of significant study. Boyce et al. (2008) showed in cow corneas that when increasing the IOP from 3.6 to 8 mmHg, 90% of the outward corneal distension at the optic axis was accounted for by deformation in the peripheral cornea. The corresponding marked difference in material parameters across the cornea (Elsheikh et al., 2013) imbues the eye with the ability to maintain focus with minimal compensation over significant variations in IOP. Given that the IOP can change due to a number of factors (see e.g. Guttridge (1994)) it would follow that there is a need for an efficient, lossless, long range elastic structure in the peripheral cornea/limbus. In other collagenous tissues that incur significant and repetitive strains, such as arteries, chordae tendinae, cartilage and adipose tissue, this structure is a network of elastic fibers (Green et al., 2014).

The structural organisation of the posterior elastic fiber system has important clinical implications for corneal transplant surgery, playing a role in providing a stable exposed transplant “bed” for implantation following lamellar keratoplasty. Given the fibers are more abundant in layers of the posterior stroma this type of surgery is likely to leave the elastic fiber system largely intact. The fiber system under normal physiological tension would provide structural and mechanical stability (Jensen et al., 2012) to the graft bed and aid donor cornea button integration. On the other hand, in penetrating keratoplasty (and other types of surgery), the elastic fiber system would be severed and the tension in the system released. The accompanying elastic recoil has potential to directly affect the peripheral cornea, the limbus, and the beams and their associated cells in the trabecular meshwork, leading to distortion of the angle, to closing of trabecular outflow channels and, in the longer term, even to glaucoma, which has long been recognised as a possible outcome of penetrating keratoplasty (see review by Al-Mahmood et al. (2012)). Since, unlike collagen and proteoglycans, elastic fibers cannot be replaced as part of matrix turnover, penetrating keratoplasty would be expected to permanently alter the mechanical properties of the cornea. This mechanism may thus need to be considered along with those currently accepted as being potential causes of surgically-induced glaucoma (Dada et al., 2008).

The elastic fiber system would be expected to play a mechanical role in the big bubble surgical technique, given that fibers appear concentrated anterior to Descemet's membrane. We suggest that the so-called “pre-Descemet's layer” is formed by the air under pressure splitting the posterior stroma along the last layer of keratocytes nearest Descemet's membrane. The separated layer, even though it is very thin, would be supported by the meshwork of elastic fibers present within the last 4–5 lamellae. Whereas collagen fibrils might slip or break under excessive extension, the elastic fibers would confer the extensibility for the so called “big bubble type 1” to withstand relatively high strains without bursting (Dua et al., 2013, Dua et al., 2015, Zaki et al., 2015, Dua et al., 2015). Peripherally, the fibers form an elastic annulus which is mainly confined to the deep limbal region. The corneolimbal annulus comprises numerous fenestrated sheets within this region and it is likely that these sheets are responsible for the circumferential air flow observed during the creation of the so-called “big bubble type 2” (Dua et al., 2013), by initially containing and restricting air to flow around the corneal perimeter.

In conclusion, we have described a complex elastic system in the posterior human cornea and limbus. This takes the form of elastic sheets in the limbus from which protrude elastic fibers into the peripheral cornea. As these fibers extend into the cornea they bifurcate and trifurcate, forming a layer of thin elastic fibers that is concentrated within the posterior 8 μm. This elastic system may have a number of developmental and structural roles. Its presence is likely to influence the biomechanical behaviour of the cornea and may provide explanations for the effects of a number of surgical interventions.

## Conflict of interest

No conflicting relationship exists for any author.

## Author contributions

PL and TW carried out the SBF SEM, making equal contributions; PL and RDY carried out the TEM. JB, TW and CPW performed the non-linear microscopy. KMM and RDY devised the project, KMM and PL drafted the manuscript, and all other authors edited this draft.
