# Supplementary figures and images for: Three-dimensional arrangement of elastic fibers in the human corneal stroma
Source: Exp Eye Res. 2016 May;146:43–53. doi: 10.1016/j.exer.2015.12.006 (PMC4889784; doi:10.1016/j.exer.2015.12.006)

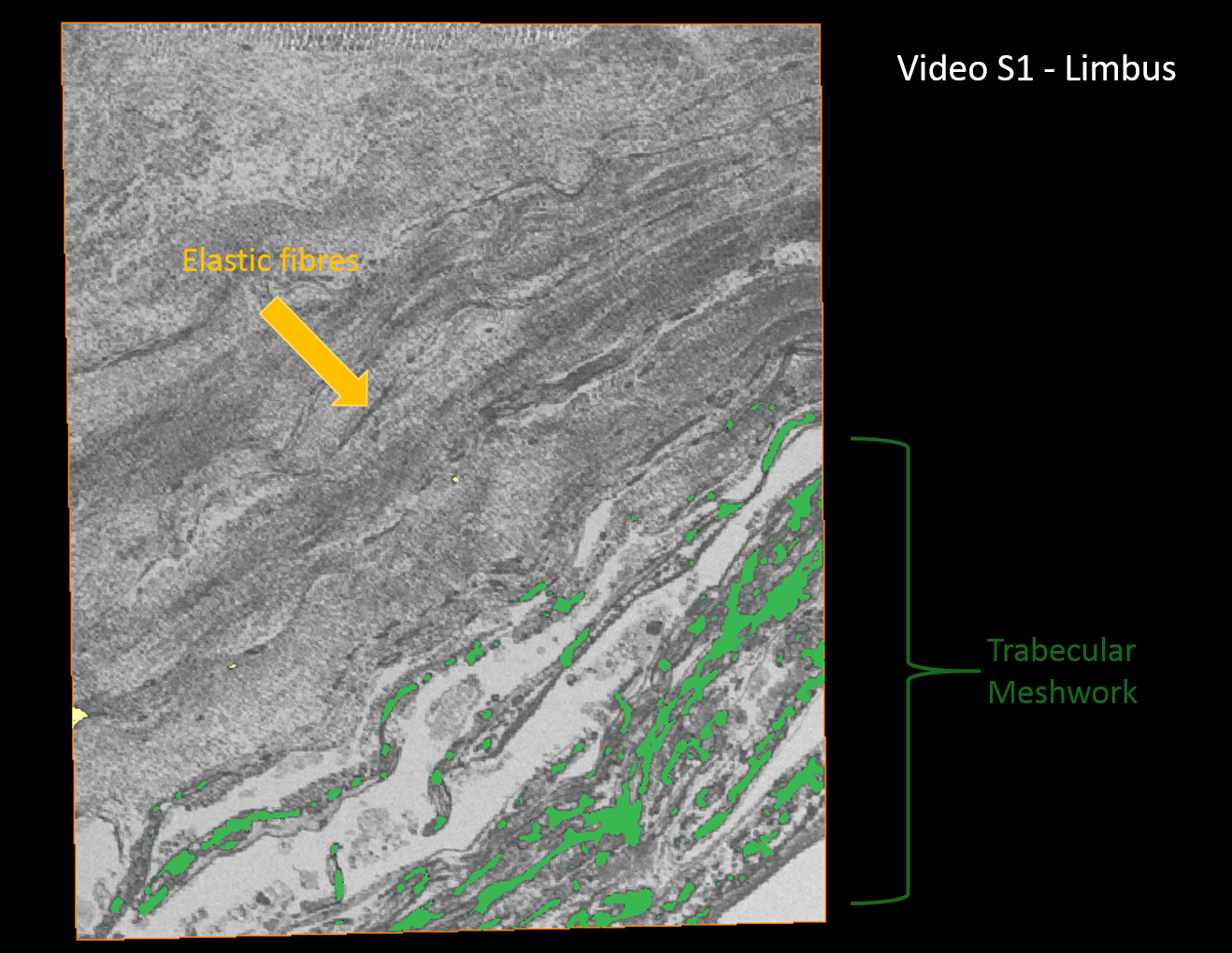

Supplement: Video clip S1 — Related to Figure 3. Rendered three-dimensional video of the corneo-limbal region near the trabecular meshwork of a cornea (Cornea 2) excised from the eye and then fixed under intraocular pressure. [file mmc1.jpg]

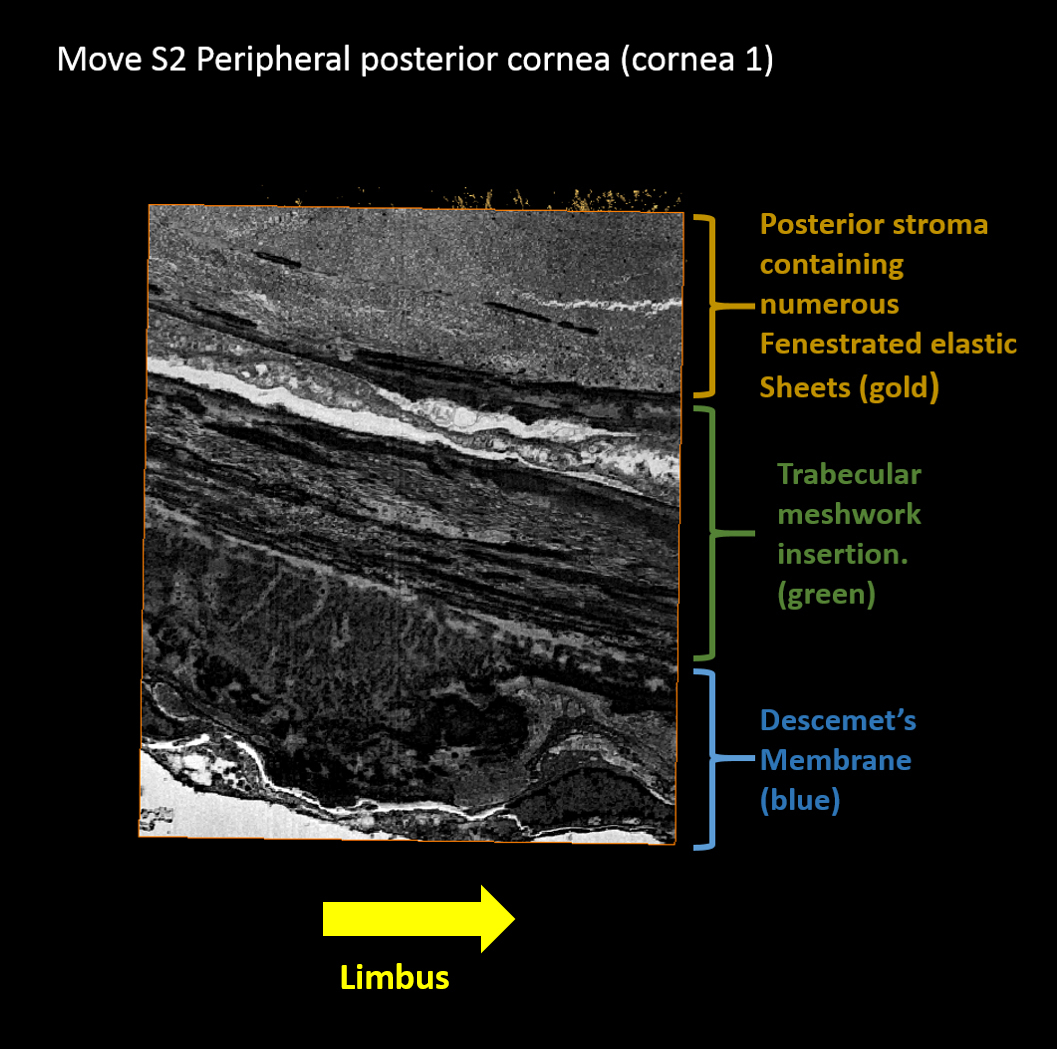

Supplement: Video clip S2 — Related to Figure 4. Rendered three-dimensional video of the corneo-limbal region of a cornea (Cornea 1) taken from a whole fixed eye. [file mmc2.jpg]

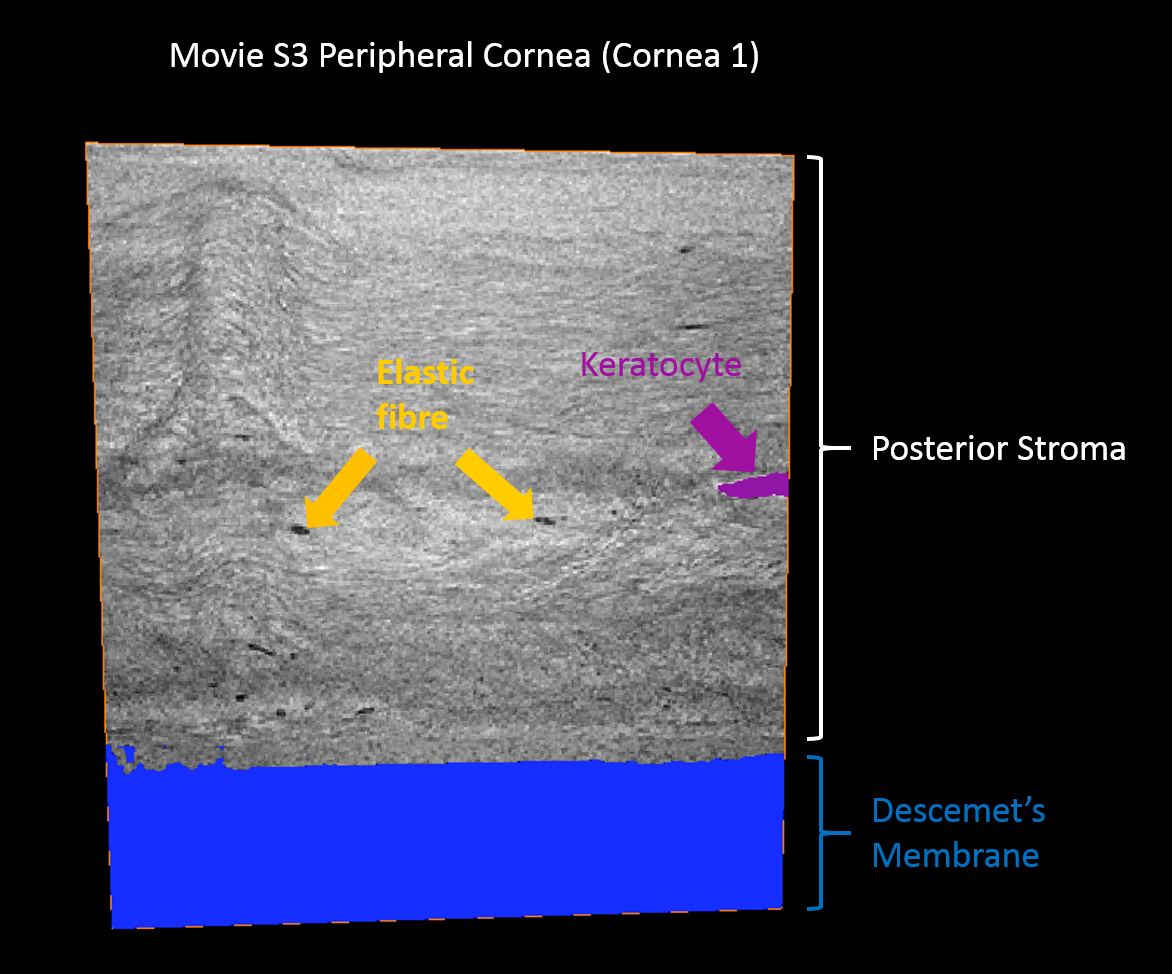

Supplement: Video clip S3 — Related to Figure 6. Rendered three-dimensional video of the peripheral posterior stroma of a cornea (Cornea 1) taken from a whole fixed eye. [file mmc3.jpg]

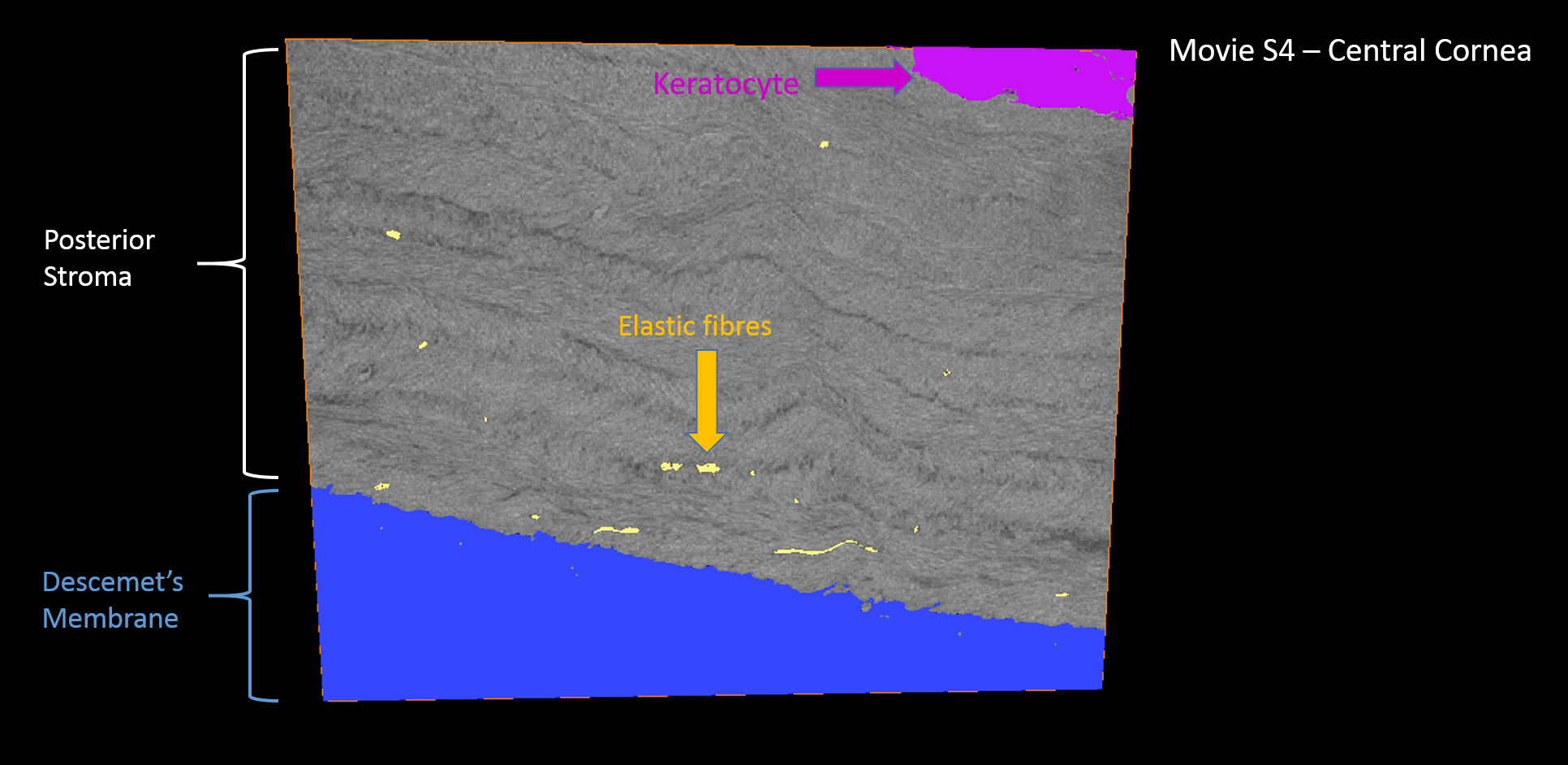

Supplement: Video clip S4 — Related to Figure 7. Rendered three-dimensional video of the central posterior cornea (Cornea 1) taken from a whole fixed eye. [file mmc4.jpg]

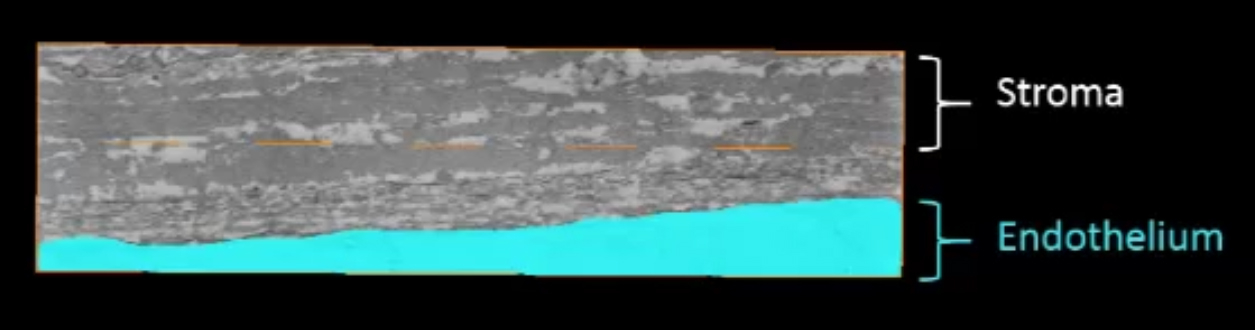

Supplement: Video clip S5 — Related to Figure 8. Rendered three-dimensional video of the central posterior corneal stroma from a 13-week-old human foetus. [file mmc5.jpg]
